# Machine-learning predicts time-series prognosis factors in metastatic prostate cancer patients treated with androgen deprivation therapy

**DOI:** 10.1038/s41598-023-32987-6

**Published:** 2023-04-18

**Authors:** Shinpei Saito, Shinichi Sakamoto, Kosuke Higuchi, Kodai Sato, Xue Zhao, Ken Wakai, Manato Kanesaka, Shuhei Kamada, Nobuyoshi Takeuchi, Tomokazu Sazuka, Yusuke Imamura, Naohiko Anzai, Tomohiko Ichikawa, Eiryo Kawakami

**Affiliations:** 1 Department of Urology, Graduate School of Medicine, Chiba University, 1-8-1 Inohana, Chuo-Ku, Chiba, Chiba 260-8670 Japan; 2 Kimitsu Chuo Hospital, Kisarazu, Chiba Japan; 3 Teikyo University Chiba Medical Center, Ichihara, Chiba Japan; 4 Department of Pharmacology, Graduate School of Medicine, Chiba University, Chiba, Chiba Japan; 5 Department of Artificial Intelligence Medicine, Graduate School of Medicine, Chiba University, Chiba, Chiba Japan; 6 Advanced Data Science Project (ADSP), RIKEN Information R&D and Strategy Headquarters, RIKEN, Kanagawa, Japan; 7 Institute for Advanced Academic Research (IAAR), Chiba University, Chiba, Chiba Japan

**Keywords:** Cancer, Oncology, Risk factors, Urology

## Abstract

Machine learning technology is expected to support diagnosis and prognosis prediction in medicine. We used machine learning to construct a new prognostic prediction model for prostate cancer patients based on longitudinal data obtained from age at diagnosis, peripheral blood and urine tests of 340 prostate cancer patients. Random survival forest (RSF) and survival tree were used for machine learning. In the time-series prognostic prediction model for metastatic prostate cancer patients, the RSF model showed better prediction accuracy than the conventional Cox proportional hazards model for almost all time periods of progression-free survival (PFS), overall survival (OS) and cancer-specific survival (CSS). Based on the RSF model, we created a clinically applicable prognostic prediction model using survival trees for OS and CSS by combining the values of lactate dehydrogenase (LDH) before starting treatment and alkaline phosphatase (ALP) at 120 days after treatment. Machine learning provides useful information for predicting the prognosis of metastatic prostate cancer prior to treatment intervention by considering the nonlinear and combined impacts of multiple features. The addition of data after the start of treatment would allow for more precise prognostic risk assessment of patients and would be beneficial for subsequent treatment selection.

## Introduction

Prostate cancer is one of the most common carcinomas, with an increasing incidence worldwide^1^. In Japan, prostate cancer was the leading cause of cancer and sixth leading cause of cancer-related deaths in 2016^2^. Deeper understanding of prostate cancer and the intrinsic function of androgens has led to the development of androgen deprivation therapy (ADT). ADT is the mainstay treatment for locally advanced and metastatic prostate cancer. ADT is also a treatment option for elderly patients with non-metastatic prostate cancer or those in poor general condition who are not candidates for surgery or radiation therapy. Prostate-specific antigen (PSA) is used as a prostate cancer-specific tumor marker that acts as a first guide and plays a key role in determining treatment efficacy of ADT. Recent reports have demonstrated that the modified Glasgow Prognostic Score (mGPS), lactate dehydrogenase (LDH) and alkaline phosphatase (ALP) levels, Eastern Cooperative Oncology Group (ECOG) performance status, and Gleason score are associated with different prognoses^3,4^.

The prognosis of prostate cancer varies considerably depending on whether the disease is non-metastatic or metastatic^5^. Many prognostic studies on metastatic castration-resistant prostate cancer (mCRPC) have been reported, while less information is available on non-castrated metastatic prostate cancer (NCMPC). Among the few reports available, a prognostic prediction model was published by Glass et al. in 2003^6^ that classified patients into three prognostic groups according to four risk factors: bone lesion localization, performance status, PSA, and Gleason score. Based on the model proposed by Glass et al., Gravis et al. proposed a prediction model^7^ that is excellent in that it only uses a single feature, ALP, which is obtained in routine clinical practice. However, the performance of the prognostic prediction model is insufficient, with a concordance index (C-index) of 0.64. To further improve prediction accuracy, it would be necessary to consider the time variation and interaction of the factors used for the prediction^8^.

Developments in computer technology have improved analytical methods for handling large-scale data, and machine learning has attracted attention also in the medical field. Machine-learning techniques are commonly used for data-driven diagnostic and prognostic predictions^9,10^. The greatest advantage of using machine learning is that it can be used to account for the combined, nonlinear effects of numerous variables and can make precise individualized predictions for heterogeneous patient populations. In recent years, machine learning-based survival analysis has been used in various carcinomas, handling many variables and enabling prognostic prediction with high accuracy^11–13^. In addition to cancer prognostic prediction, there are many other areas where machine learning can contribute to biomedical research, such as drug interaction analysis^14,15^.

Therefore, the purpose of this study was to develop a clinically applicable prognostic prediction model for prostate cancer treated with androgen deprivation therapy based on multiple features using machine learning. We then additionally examined the impact on prediction accuracy of incorporating features after the start of treatment. To ensure applicability in clinical practice, this study used features obtained routinely in medical practice, such as peripheral blood sampling and urinalysis.

## Result

### Patient background

This study included 340 patients with prostate cancer. Of these, 30 patients who had started treatment at other hospitals were excluded (Fig. S1). A final total of 310 patients were included in the study, comprising 207 and 103 patients in the training and test cohorts, respectively. The median age was 74 years, and the median initial PSA level was 40.365. The rates of Gleason score ≥ 8 was 54.2%. The rate of metastasis was 41.6% (Table 1). No significant differences were observed between the training and test cohorts in patient backgrounds. Among the 36 features used as explanatory variables, only uric acid (UA) was significantly different between the training and test cohorts (Table 2).

**Table 1:** Clinical backgrounds of 310 patients with prostate cancer.

Background	All patients (N = 310)	Training cohort (N = 207)	Test cohort (N = 103)	*P* value
Age, years (range)	74 (46–93)	74 (46–90)	74 (48–93)	0.2617
Initial PSA, ng/dL (range)	40.365 (0.19–13,050)	39.31 (2.05–13,050)	42.55 (0.19–6421.08)	0.6853
Gleason score ≥ 8, n (%)	168 (54.2)	116 (56)	52 (50.5)	0.3748
T ≥ 3, n (%)	193 (62.3)	128 (61.8)	65 (63.1)	0.8995
N+, n (%)	81 (26.1)	52 (25.1)	29 (28.2)	0.5848
M+, n (%)	129 (41.6)	87 (42)	42 (40.8)	0.9028

T ≥ 3 means tumor stage 3 or greater, N+ means lymph node metastasis, M+ means metastasis.

**Table 2:** Characteristics of analysis factor.

Factor	All patients (N = 310)	Training cohort (N = 207)	Test cohort (N = 103)	*P* value
Age (years)	74 (46–93)	74 (46–90)	74 (48–93)	0.2617
Initial PSA (ng/dL)	40.365 (0.19–13,050)	39.31 (2.05–13,050)	42.55 (0.19–6421.08)	0.6853
AST (U/L)	22 (11–129)	22 (12–95)	23 (11–129)	0.9234
ALT (U/L)	17 (5–102)	17 (5–102)	17 (5–80)	0.9014
LDH (U/L)	193.5 (82–4621)	197 (119–833)	192 (82–4621)	0.2807
GTP (U/L)	32 (9–358)	32.323 (10–215)	31.703 (9–358)	0.2882
TP (g/dL)	7 (5.1–8.6)	7 (5.1–8.4)	6.9 (5.1–8.6)	0.5483
Alb (g/dL)	4.1 (2.5–4.9)	4.1 (2.5–4.9)	4.1 (2.6–4.8)	0.9776
UA (mg/dL)	5.8 (2.2–12)	5.727 (2.2–9.2)	6 (3.4–12)	0.0414
UN (mg/dL)	16 (5–58)	16 (5–58)	16 (8–33)	0.4691
CRE (mg/dL)	0.84 (0.52–8.02)	0.84 (0.52–8.02)	0.84 (0.59–2.01)	0.417
Tbil (mg/dL)	0.7 (0.2–2.8)	0.7 (0.2–2.8)	0.7 (0.3–2.3)	0.4782
Dbil (mg/dL)	0.1 (0–0.3)	0.1 (0–0.3)	0.1 (0–0.3)	0.7917
TCHO (mg/dL)	186.2185 (101–303)	185 (101–275)	187.591 (119–303)	0.056
TG (mg/dL)	125.2795 (45–912)	121 (47–912)	136.711 (45–285)	0.8275
Ca (mg/dL)	9 (6.7–11.6)	9 (7.7–10.7)	8.9 (6.7–11.6)	0.6029
Na (mmol/L)	140 (130–146)	140 (130–146)	140 (132–144)	0.9252
K (mmol/L)	4.21845 (3.1–6.6)	4.2 (3.1–6.6)	4.3 (3.1–5.4)	0.2421
Cl (mmol/L)	106 (95–116)	106 (96–116)	105.693 (95–111)	0.8685
WBC (× 10^3^/μL)	6.2 (2.4–19.7)	6.2 (2.4–12.8)	6.3 (2.5–19.7)	0.4734
RBC (× 10^6^/μL)	4.33 (1.93–6.49)	4.35 (1.93–6.1)	4.29 (2.82–6.49)	0.9218
Hb (g/dL)	13.5 (5.5–18.3)	13.5 (5.5–17.6)	13.5 (8.1–18.3)	0.8833
HCT (%)	40 (16.5–53.8)	39.8 (16.5–51.9)	40.2 (24.3–53.8)	0.7497
MCV (fL)	92.4 (72.3–114)	92.3 (75.9–114)	92.9 (72.2–110)	0.6529
MCH (pg)	31.1 (22.5–38.1)	31.1 (24.6–37.5)	31.1 (22.5–38.1)	0.9092
MCHC (%)	33.6 (30.8–36.3)	33.6 (30.8–36.3)	33.6 (30.8–36.2)	0.4757
PLT (× 10^3^/μL)	206 (18–466)	205 (18–466)	211 (84–433)	0.5377
ALP (U/L)	247.5 (110–9481)	248 (110–9481)	246 (123–2469)	0.6496
PT (s)	11.4541 (9.9–20.4)	11.4022 (9.9–19.6)	11.66675 (10–20.4)	0.3152
PTINR	1.00371 (0.9–1.88)	1.00321 (0.9–1.76)	1.006855 (0.9–1.88)	0.6916
BS (mg/dL)	113.178 (74–282)	115.544 (74–282)	107.5 (86–213)	0.0984
CHE (U/L)	287 (112–539)	287 (115–539)	286.5 (112–468)	0.5798
CRP (mg/dL)	0.2 (0–24.9)	0.2 (0–24.9)	0.16444 (0–8.8)	0.1746
UpH	6 (5–8)	6 (5–8)	6 (5–8)	0.7532
URBC (/HPF)	1 (0–50)	1 (0–50)	1 (0–50)	0.7784
UWBC (/HPF)	1 (0–50)	1 (0–30)	1 (0–50)	0.2623

The data in the brackets indicate a range of values.

### Prognostic prediction at the start of treatment

**Figure 1:**
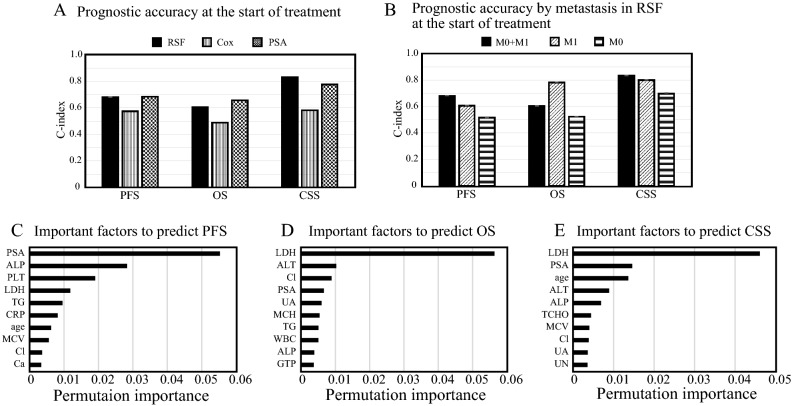
Comparison of accuracy of prognostic prediction models. (**A**) Comparison of C-index for each prognostic prediction model. Black, shaded, and horizontal bars indicate RSF, Cox proportional hazards, and PSA models, respectively. (**B**) Comparison of C-index for application of RSF model to patients with metastatic and non-metastatic prostate cancer. Black, striped, and dotted bars indicate all patients with prostate cancer, patients with metastatic prostate cancer, and patients with non-metastatic prostate cancer, respectively. (**C** to **E**) Permutation importance in prediction of progression (**C**), overall survival (**D**), and cancer specific survival (**E**) based on the RSF model.

To evaluate the usefulness of multiple variables for predicting prostate cancer prognosis, 36 features including age, peripheral blood tests, and urinalysis were used in the analysis. To maintain impartiality among models and avoid multicollinearity among features, the variables were first selected using RSF based on permutation importance calculated in the training cohort. Selected top important variables with positive permutation importance were used in subsequent RSF and Cox proportional hazards analyses. In addition, we created a prediction model for PSA (a tumor marker for prostate cancer) alone and compared its accuracy using the C-index (Fig. 1A). The C-indices for prediction in test cohort using the Cox proportional hazards model were 0.573, 0.488, and 0.582 for PFS, OS, and CSS, respectively. The corresponding C-indices for prediction using PSA alone were 0.684, 0.656, and 0.774, respectively. Finally, the corresponding mean C-indices (standard deviation) with RSF were 0.681 (0.002), 0.603 (0.005), and 0.832 (0.004), respectively. In terms of prediction at the start of treatment, the conventional prediction using PSA was almost as accurate as the RSF in predicting PFS, OS, and CSS, respectively. Next, we calculated the prognostic accuracy of the RSF model created above when applied separately to metastatic and non-metastatic prostate cancer patients. The results revealed improved OS prediction accuracy in metastatic prostate cancer, while, for non-metastatic tumors, predictive performance was poor for all predictions (Fig. 1B). We identified PSA as an important predictor in RSF for predicting PFS and LDH as an important predictor of OS and CSS (Fig. 1C–E).

### Prognostic predictions considering temporal changes after the start of treatment

**Figure 2:**
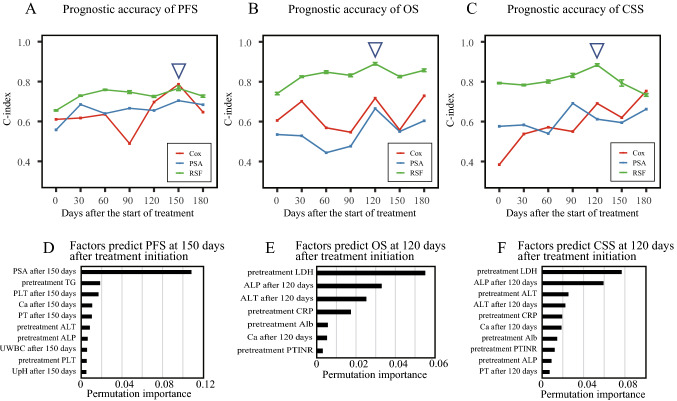
Time-series of prognostic accuracy for patients with metastatic prostate cancer. (**A** to **C**) Accuracy of prediction of progression (**A**), overall survival (**B**), and cancer specific survival (**C**). The green, red, and blue lines indicate the RSF, Cox proportional hazards, and prognostic PSA-based models, respectively. The triangle mark indicates the time point at which prediction accuracy was the highest for RSF prediction. Error bars represent standard deviations of 10 independent RSF. Permutation importance in prediction of progression at 150 days after treatment initiation (**D**), overall survival at 120 days after treatment initiation (**E**), and cancer-specific survival at 120 days after treatment initiation (**F**). The number of factors was defined as the top 10 factors or those with positive importance.

We further aimed to improve the prediction of metastatic prostate cancer by considering post-treatment changes. Patients with metastatic prostate cancer were assigned to the same training and test cohort as in the pretreatment analysis. In this analysis, the C-indices of the Cox proportional hazards model and prediction model using only PSA were calculated for comparison with the RSF model (Fig. 2). For predicting OS and CSS, the RSF model was more accurate than the other models: for the RSF model, it had the highest C-index (standard deviation) for predicting PFS at 150 days post-treatment at 0.766 (0.011), and at 120 days post-treatment the C-index for predicting OS and CSS were 0.89 (0.006) and 0.883 (0.006), respectively. The Cox proportional hazards model and RSF had similar predictive performance in predicting PFS at 150 days after treatment initiation. On the other hand, the prediction performance of RSF was appreciably better than the other two models in predicting OS and CSS. Compared to the other prognostic prediction models, the RSF forecasting model tended to have less variation in forecast accuracy depending on the time of year. While RSF was able to predict prognosis for metastatic prostate cancer with relatively high accuracy, it was difficult to predict prognosis for non-metastatic prostate cancer with high accuracy (Fig. S2). In this prognostic analysis of metastatic prostate cancer patients, the addition of the Gleason score, an important pathologic factor in prostate cancer, as a predictor did not result in a notable improvement in prognostic accuracy (Fig. S3). The distribution of Gleason scores in patients with metastatic prostate cancer is shown in Table S1.

### Permutation importance in RSF analysis

Feature importance can be used to explain the contribution of explanatory variables in machine learning predictions^16^. We used permutation importance, a type of feature importance, to evaluate the contribution of explanatory variables in the RSF. Permutation importance at the time of prediction when the C-index was maximum in each of the RSF analyses described above is presented in Fig. 2D–F. For PFS prediction at 150 days after the start of treatment, the most important variable was PSA after treatment. For the prediction of OS and CSS at 120 days after the start of treatment, the most important factors were LDH before treatment and ALP after treatment. For both OS and CSS prediction, PSA levels before and after treatment were not included as an important predictor.

### Construction of survival trees based on RSF

**Figure 3:**
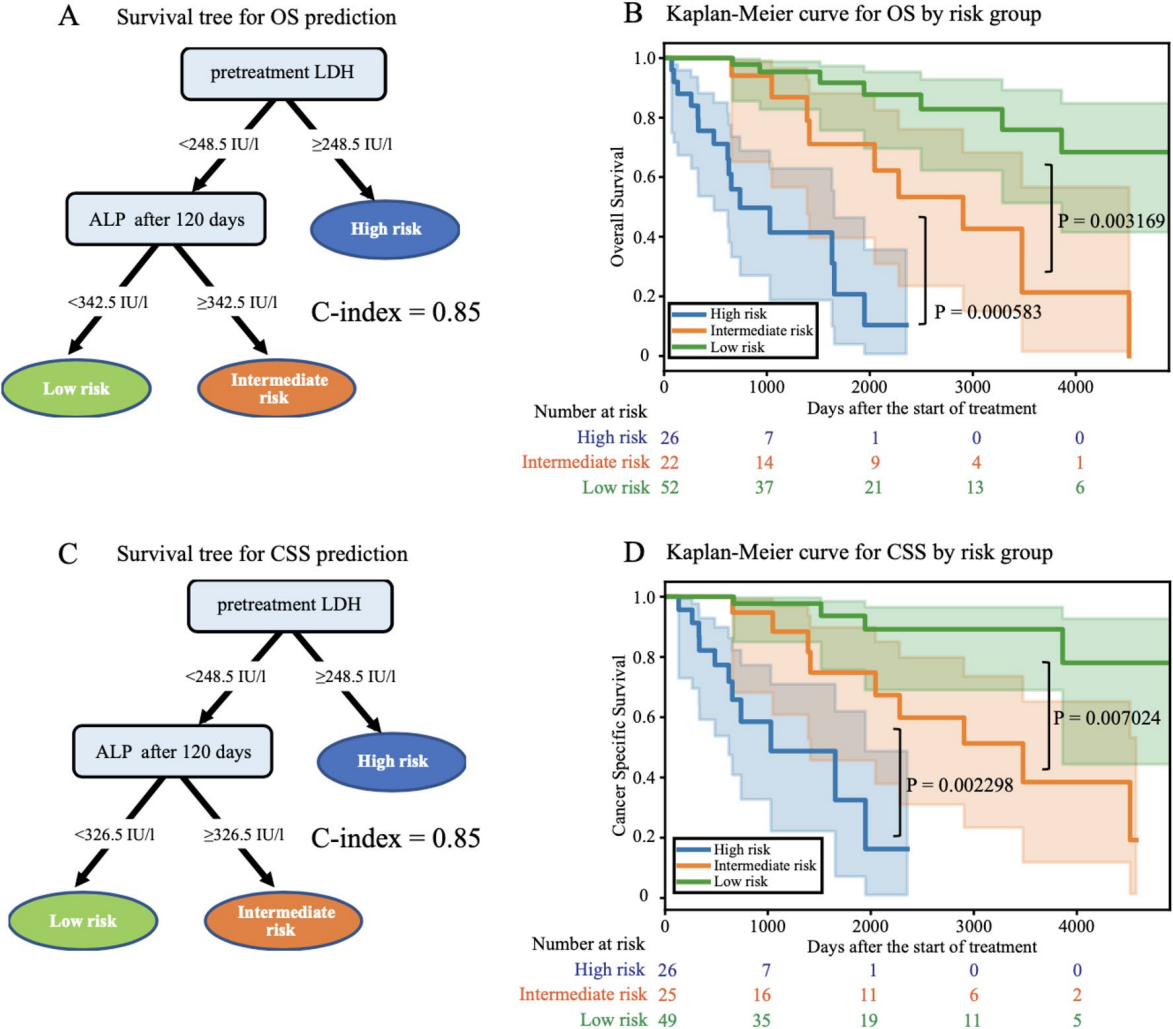
Survival tree predicting overall survival (**A**) and cancer-specific survival (**C**). Kaplan–Meier curves of survival tree prognostic classification results for overall survival prediction (**B**) and cancer-specific survival prediction (**D**). P-values were calculated by the log-rank test.

As described in the previous section, prognosis prediction using RSF exhibited excellent accuracy. However, since RSF is an ensemble learning method with multiple survival trees and requires many explanatory variables, it is not easy to use it for prognostic prediction in real clinical practice. Therefore, we constructed a simplified survival tree model with a few most important variables in the RSF model. Since the contribution of post-treatment PSA was predominantly large in predicting PFS prognosis, and the benefit of combining multiple variables by survival tree was limited, we focused only on OS and CSS and constructed a survival tree model based on the top five important variables in the RSF models at 120 days after the start of treatment. The obtained survival trees predicting OS and CSS both consisted of LDH before treatment initiation and ALP 120 days after the start of treatment (Fig. 3A,C). The cut-off values of pre-treatment LDH and post-treatment ALP in the prediction models of OS and CSS were 248.5 IU/L and 342.5/326.5 U/L, respectively. The C-index for prediction accuracy was 0.85 for both OS and CSS. Based on these survival trees, three patient populations were identified that were associated with OS and CSS prognosis: the first was a very poor prognosis population with high preoperative LDH (> 248.5 IU/L), in which about 70% of patients would die within 5 years; the population with LDH < 248.5 IU/L was further divided into two groups based on post-treatment ALP. The group with high ALP is at intermediate risk and has a 5-year survival rate of about 70%. The population with low LDH before treatment and low ALP after treatment had a very good prognosis, with a 5-year survival rate exceeding 90% (Fig. 3B,D).

## Discussion

Compared to conventional statistical analysis, machine learning can handle a large number and variety types of variables, and the machine can automatically learn and discover rules and patterns underlying the data. Various analyses using machine learning have been reported to improve the diagnostic rates of imaging and biopsy tests for prostate cancer^17,18^. However, prognostic analyses using machine learning for ADT remain scarce. In this study, we developed an approach to predict the prognosis of metastatic prostate cancer treatment over time: at the start of treatment and after the start of treatment. Pre-treatment and post-treatment features were combined to achieve a more accurate prediction.

We attempted to predict prognosis for both non-metastatic and metastatic prostate cancer, but it was difficult to predict prognosis in patients with non-metastatic prostate cancer (Fig. S2). The RSF model at the start of treatment showed improved predictive accuracy in metastatic prostate cancer patients, while it showed decreased accuracy in non-metastatic prostate cancer patients. This may be due to the fact that non-metastatic prostate cancer patients in this study had a smaller proportion of cancer deaths than metastatic prostate cancer patients, and included more senility and death from other causes, which are difficult to predict from clinical laboratory data. Moreover, prognostic factors for non-metastatic prostate cancer are limited, with only a few factors, such as PSA doubling time, reported in the literature^19–21^. Therefore, we focused on predicting the prognosis of metastatic prostate cancer. In this study, the RSF model was more accurate than other models in predicting OS and CSS in time-series metastatic prostate cancer. On the other hand, there was no significant difference in PFS prediction. First, the reason for the lack of significant difference in PFS prediction accuracy may be that factors other than PSA were less important in predicting PFS, since the definition of relapse in this study was biological relapse, which was defined as an increase in PSA. Second, the reason for the superior accuracy of the RSF model in predicting OS and CSS could be that parameters other than PSA are important as predictors in predicting OS and CSS, as shown by the results of Permutation Importance. Furthermore, regarding the difference between the RSF model and the Cox proportional hazards model, the RSF model may have been able to make more accurate predictions for many parameters in terms of its ability to make nonlinear predictions. However, since over-fitting should also be considered in this respect, we believe that validation using external data will be necessary in the future. Regarding the tumor marker PSA, our previous study reported no difference in OS according to initial PSA levels in patients with metastatic prostate cancer^22^. For prognostic factors other than PSA, the modified Glasgow Prognostic Score (mGPS), Eastern Cooperative Oncology Group (ECOG) performance status, LDH, ALP, and Gleason Score have been reported as prognostic factors for metastatic prostate cancer^3,4^. Among Japanese patients with de novo metastatic prostate cancer, LDH and C-reactive protein (CRP) have been reported as independent risk factors for OS in analyses identifying true high-risk groups that meet the CHAARTED or LATITUDE criteria^23^. Several studies support the results of this study. However, these were all prognostic analysis based on data at the start of treatment and did not include post-treatment changes. While prognostic predictions based on data at the start of treatment are important, the course of treatment affects the prognosis, and in some cases the actual prognosis differs from the initial risk assessment. To identify such cases and enable a more accurate prognosis, it is necessary to add post-treatment data as predictors and to update the prediction. In this study, we could first identify the poor prognosis group based on LDH at the start of treatment for both OS and CSS, and further classified the remaining patients into two groups with different prognoses using ALP after the start of treatment. This suggests that additional risk assessment during the course of treatment, in addition to risk classification at the start of treatment, can provide a more accurate prognosis. From a pathological perspective, we used the Gleason score in the RSF analysis, which has been used in existing risk classifications, but this did not clearly improve the C-index. Patients with metastatic prostate cancer tend to have high Gleason scores, and in fact, Gleason score ≥ 8 accounted for more than 70% of the patients in this case group.

Gravis et al. reported a prediction model for NCMPC based on the prediction model proposed by Glass et al.^7^. They claimed that ALP levels at the start of treatment (normal vs. abnormal) were the strongest predictor of OS. This prediction model had a C-index of 0.64, was simpler than the prediction model developed by Glass et al., and exhibited comparable performance. The C-index of the model reported by Gravis et al. was 0.72 in the analysis using the data in this study. The C-index for our RSF model in this study using the data at the start of treatment was 0.74. Although our RSF model was only slightly more accurate than the previously reported model, the C-index was improved to 0.85 in this study by creating an algorithm using a survival tree with the addition of time-series data. The new algorithm for metastatic prostate cancer we have created based on the survival tree made predictions using two variables (pre-treatment LDH and post-treatment ALP) with a C-index of 0.85, which was higher than the accuracy of previous prediction models. LDH and ALP values can be obtained from routine blood tests and can be used for time-series evaluation.

Our study had several limitations. First, it was a retrospective analysis with a limited number of cases at a single institution, and there may have been a selective bias. In general, machine learning methods divide datasets into training and test data, create a prediction model with the training data, and evaluate the model using the test data. If the number of cases is small, a biased prediction model (overfitting) may be created if the training data have extreme characteristics. We used data from 129 patients with metastatic prostate cancer for the training in our analysis. To increase variation in the training data and suppress overfitting, we intend to conduct further analysis using larger-scale data from multiple institutions in the future. In this study, we performed random data splitting. Although there were no significant differences between the train cohort and the test cohort, it is necessary to consider the use of data splitting methods such as cross validation in future analyses to create a new model. Second, because we defined progression as biological progression caused by elevated PSA levels, post-treatment PSA inevitably became the most important factor for predicting progression. Future research should focus on clinical progression, such as disease worsening on imaging and the appearance of new metastases.

In conclusion, this study demonstrated that machine learning and combined assessment of pre- and post-treatment variables were useful for creating an accurate prognostic prediction model for ADT in metastatic prostate cancer. This result may be harnessed as a new evaluation index for the treatment of metastatic prostate cancer.

## Methods

### Patient selection and analysis factors

This retrospective study included 340 patients with prostate cancer who received ADT as an initial treatment between 1996 and 2019 at the Department of Urology, Chiba University Hospital. Of these, 30 patients who had started treatment at other hospitals were excluded. The dataset was randomly divided into training and test cohorts. In total, 207 and 103 patients were classified into the training and test cohorts, respectively. We first analysed 36 features before treatment including age at diagnosis, peripheral blood sampling, and urinalysis to examine their association with progression-free survival (PFS), overall survival (OS), and cancer-specific survival (CSS). An additional analysis focusing on patients with metastatic prostate cancer was performed, which considered data at the start of treatment as well as subsequent changes. In the analysis, 35 features after the start of treatment including peripheral blood sampling and urinalysis were combined with the 36 pretreatment features and used for prediction. This study was conducted in accordance with the ethical principles of the Declaration of Helsinki. This retrospective study of clinical information was approved by the Ethics Committee of Chiba University (Institutional Review Board (IRB) no. M10238). The IRB waived the requirement for written consent in this study due to the retrospective nature of data collection.

### Survival analysis

We employed random survival forests (RSF) for machine-learning survival analysis. The rationale for this is as follows. First, Random forests and derivatives outperform other machine learning methods in predictions using clinical laboratory values^24,25^. Secondly, RSF is implemented within scikit-survival, making it easy to calculate variable importance and transfer it to the survival tree model, which is also implemented in scikit-survival. Finally, like random forests, RSFs are suitable for variable selection because they selectively use a small number of variables^26^. RSF is a nonlinear survival model that combines ensemble learning and decision tree^27^. In RSF, multiple sets of data termed bootstrap samples are created. At each node of the survival tree, feature and its threshold value were determined such that the difference in hazard function between cases separated by the nodes was maximized. The ensemble hazard function of each patient was estimated by averaging the hazard functions of multiple trees created in this manner. In this study, RSF was used to predict PFS, OS, and CSS. Analysis was performed using scikit-survival Python package. We ran sksurv.ensemble.RandomSurvivalForest with the default parameters, except for the following parameters; n_estimators = 2000, min_samples_split = 10, min_samples_leaf = 15. The reason for using nearly default parameters is that hyperparameter optimization under limited training data conditions may result in lower accuracy, and random forests are robust to hyperparameter changes^28^. Since RSF uses bootstrap samples, the value of the estimated survival function varies slightly with each run. Therefore, we ran the RSF 10 times independently and used the average C-index as the prognostic performance indicator. We calculated permutation importance to evaluate the contribution of explanatory variables to RSF prediction performance. The permutation importance indicates the change in predictive performance (AUC in this case) when an explanatory variable is randomly shuffled, with a positive importance indicating that the variable is necessary for prediction and a negative importance indicating that using the variable reduces predictive performance^29^. For example, if the AUC drops by 0.05 when a variable is randomly shuffled, the permutation importance score for that variable is 0.05. Permutation importance was calculated using eli5 Python package.

A Cox proportional hazards model was used as the conventional statistical survival analysis for comparison. To make the conditions fair across models, the variables were selected based on the permutation importance calculated by RSF pretraining, and the same variables were used in the Cox proportional hazards model.

### Survival tree

A survival tree represents the individual tree comprising the aforementioned RSF. This method analyses data using a tree diagram and exhibits excellent semantic interpretability in that it visualizes the classification criteria, facilitating comprehension of the results^30^. RSF can calculate feature importance during classification. By integrating the results of multiple survival trees, RSF allows highly accurate predictions for individual patients, but make it difficult for humans to interpret the predictive results and rationale. In this regard, survival tree may be a better solution for clinical implementation. In this study, we developed survival trees for OS and CSS using the top five important features obtained in the RSF analysis. We used the Optuna Python package to optimize the parameters of survival tree to achieve the highest prediction rate in training cohort^31^.

### Missing value imputation

To compensate for missing values in the dataset used in this study, we used the missForest algorithm implemented in R^32^. MissForest is a non-parametric imputation method that uses a random forest which can learn nonlinear relationship between variables, easily handle mixed-type data, and calculate out-of-bag (OOB) errors. First, the average value was used to tentatively fill the missing values, and the random forest was then repeatedly applied to predict the missing parts. Stekhoven et al. reported that missForest was superior to other widely used imputation algorithms such as KNNimpute, MICE, and MissPALasso.

### Evaluation of survival model accuracy

The predictive performance of the survival models, including RSF, Cox proportional hazards model, and survival tree, was evaluated using the Harrell's concordance index (C-index). The C-index is a generalization of the area under the ROC curve (AUC) that considers censored data^33^. This represents an assessment of the discriminatory power of the model, which is the ability of the model to correctly provide a ranking of survival times for each patient based on hazard function. Time-dependent ROC analysis is another method of evaluating prediction accuracy in survival analysis. However, we adopted the C-index to express the transition of prediction accuracy in the time-series analysis in an easily understandable manner, given the need for analysis at multiple time points after the start of treatment.

### Statistical analysis

The Kaplan–Meier method was used to generate survival curves to evaluate survival probability of given groups. Statistical difference in the survival probabilities between groups was assessed using log-rank test. For the analysis of the training and test cohorts, Welch’s t-test and Fisher’s exact test were used for continuous and categorical variables, respectively. Statistical analysis was performed using JMP^®^ 15.2. The significance level for each test was set at α = 0.05.

## Supplementary Information

Supplementary Information.

## Data Availability

The datasets generated and analysed during the current study are not publicly available due to ethical regulations because the data contain personal information but are available from the corresponding author on reasonable request.
