# Supplementary material for: Machine-learning predicts time-series prognosis factors in metastatic prostate cancer patients treated with androgen deprivation therapy
Source: Sci Rep. 2023 Apr 18;13:6325. doi: 10.1038/s41598-023-32987-6 (PMC10113215; doi:10.1038/s41598-023-32987-6)
Supplement: Supplementary file 1 — Supplementary Information. [file 41598_2023_32987_MOESM1_ESM.pdf]

Supporting Information

Title

Machine-learning predicts time-series prognosis factors in metastatic prostate cancer patients treated with androgen deprivation therapy

Shinpei Saito[1, 5], *Shinichi Sakamoto[1], Kosuke Higuchi[2], Kodai Sato[1, 5], Xue Zhao[1], Ken Wakai[3], Manato Kanesaka[1], Shuhei Kamada[1], Nobuyoshi Takeuchi[1], Tomokazu Sazuka[1], Yusuke Imamura[1], Naohiko Anzai[4], Tomohiko Ichikawa[1], Eiryo Kawakami[5, 6, 7]

[1] Department of Urology, Graduate School of Medicine, Chiba University

[2] Kimitsu Chuo Hospital

[3] Teikyo University Chiba Medical Center

[4] Department of Pharmacology, Graduate School of Medicine, Chiba University

[5] Department of Artificial Intelligence Medicine, Graduate School of Medicine, Chiba University

[6] Advanced Data Science Project (ADSP), RIKEN Information R&D and Strategy Headquarters, RIKEN

[7] Institute for Advanced Academic Research (IAAR), Chiba University

*Corresponding author

	All patients (N=129)	Training cohort (N=87)	Test cohort (N=42)	*P* value
**Gleason score (%)**				
6 or less	0 (0)	0 (0)	0 (0)	-
7	13 (10.1)	8 (9.2)	5 (11.9)	0.5448
8	35 (27.1)	25 (28.7)	10 (23.8)	0.8274
9 or greater	66 (51.2)	45 (51.7)	21 (50.0)	1
unknown	15 (11.6)	9 (10.3)	6 (14.3)	0.5628

Table S1. The distribution of Gleason scores of 129 patients with metastatic prostate cancer

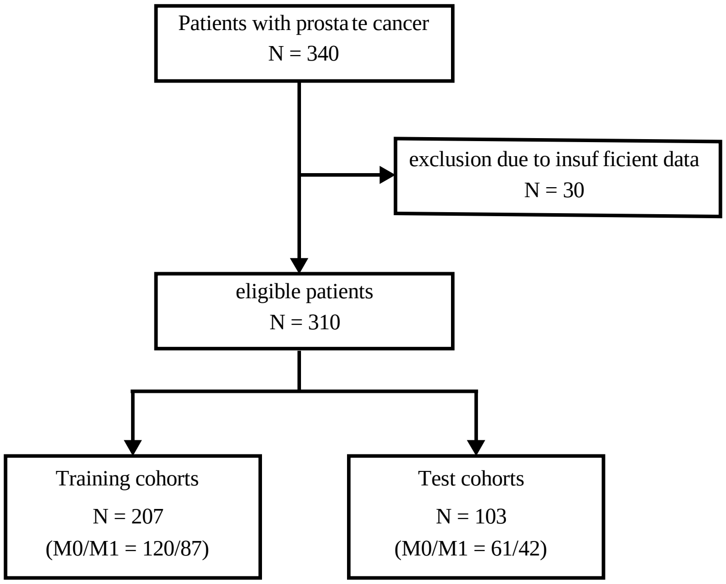

Figure S1. Patient’s selection protocol.

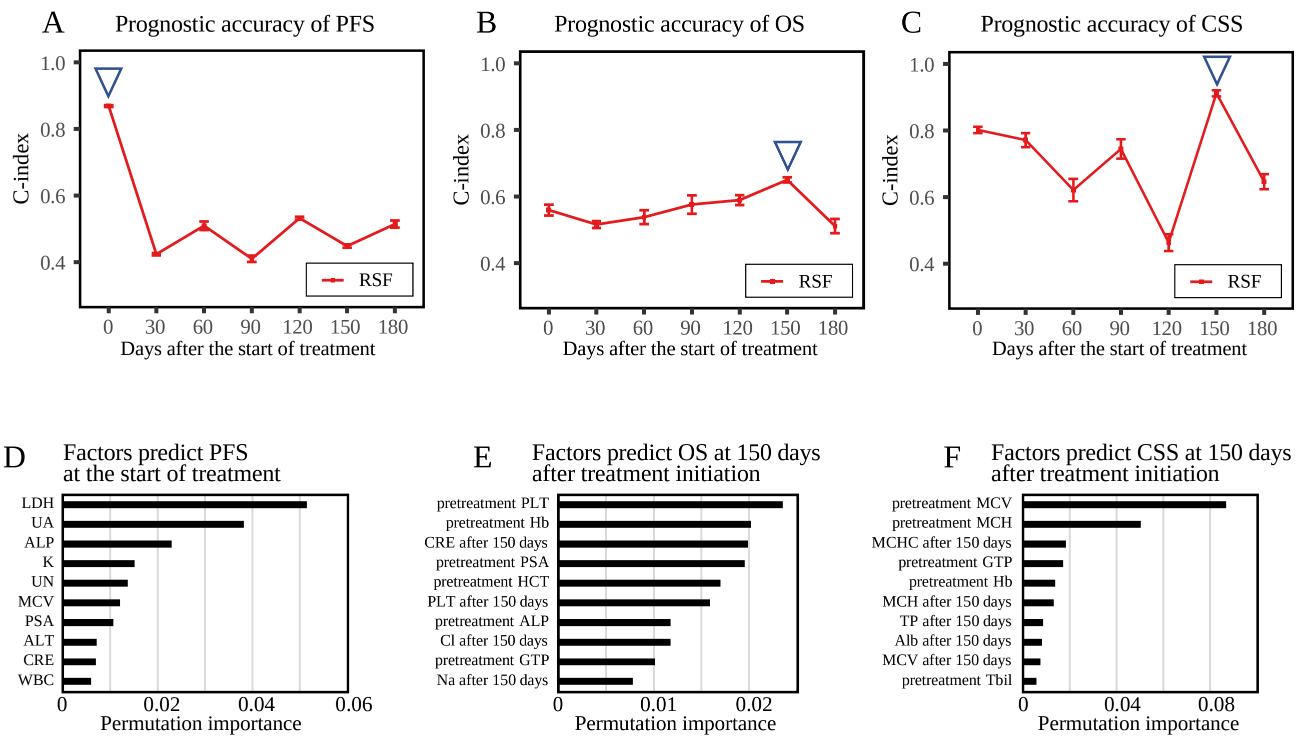

Figure S2. Time series of prognostic accuracy for patients with non-metastatic prostate cancer. Accuracy of prediction of progression (A), overall survival (B), and cancer-specific survival (C). The triangle mark indicates the time point at which prediction accuracy was the highest for the RSF prediction. Error bars represent standard deviations of 10 independent RSF. Permutation importance in prediction of progression at treatment initiation (D), overall survival at 150 days after treatment initiation (E), and cancer-specific survival at 150 days after treatment initiation (F). The number of factors was defined as the top 10 factors or those with positive importance.

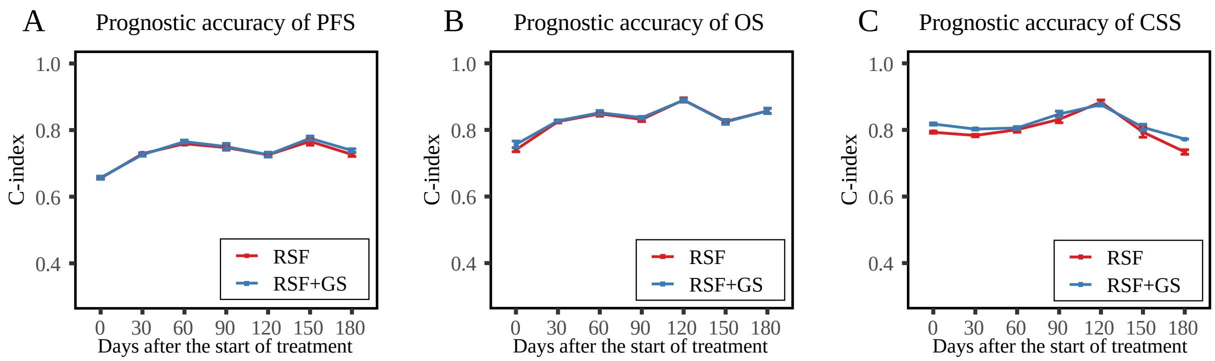

Figure S3. Comparison of C-index in two prognostic prediction models. Accuracy of prediction of progression (A), overall survival (B), and cancer-specific survival (C). The red and blue lines indicate the C-indices of the RSF model and model with Gleason score added as a predictor to the RSF model, respectively. Error bars represent standard deviations of 10 independent RSF.
